# The Effect of a Modified GH11 Xylanase on Live Performance, Gut Health, and *Clostridium perfringens* Excretion of Broilers Fed Corn-Soy Diets

**DOI:** 10.3389/fvets.2021.678536

**Published:** 2021-06-07

**Authors:** Basheer Nusairat, Jeng-Jie Wang

**Affiliations:** ^1^Department of Animal Production, College of Agriculture, Jordan University of Science and Technology, Irbid, Jordan; ^2^BioResource International, Inc., Durham, NC, United States

**Keywords:** xylanase, performance, lesion score, digestibility, broiler

## Abstract

Xylanase enzymes and other feed additives are being used more commonly in poultry feed to reduce feed cost, improve performance, and maintain gut health. Five corn-soy-based dietary treatments were designed to compare the effect of different inclusion levels of high-efficiency GH11 xylanase on live performance, gut lesions, and *Clostridium perfringens* excretion in littler samples of broiler chickens. Diets were the standard diet (positive control; PC); a diet of reduced energy by 130 kcal/kg diet (negative control; NC); NC with xylanase at 10 XU/g of feed (NC + 10); NC with xylanase at 12.5 XU/g of feed (NC + 12.5); NC with xylanase at 15 XU/g of feed (NC + 15). Data were analyzed with one-way ANOVA. At 42 d, birds fed NC + 12.5 and NC + 15 were heavier (*P* < 0.05) than NC and comparable improvement to birds fed PC. Significant Improvement in FCR (*P* = 0.0001) was observed from 1 to 42 d for NC + 12.5 and NC + 15 compared with NC. Supplementation of xylanase reduced (*P* < 0.005) 21 d intestinal lesion score at 21 d with further improvement (*P* < 0.0001) at 42 d. NC + 15 reduced lesion scores by 24% compared with NC. Xylanase supplementations reduced litter *C. perfringens* cell forming unit per gram (CFU/g) compared with NC with the highest reduction of NC + 15 treatment by ~27%. In conclusion, xylanase can be included in reduced-energy diets up to 15 XU/g of feed to improve live performance, energy digestibility, and reduce intestinal lesion scores in broilers.

## Introduction

Providing adequate quantities of animal protein to meet the demands of a continuously growing population requires that livestock producers must increase the production efficiency of farm animals. This can be achieved through improved genetics, management, and nutrition. Furthermore, feed ingredient prices continue to increase, resulting in minimizing the profit for the producers and forcing them to seek cheaper alternative ingredients, typically of lower quality and higher fiber content (1).

Feed composition plays an important role in efficiently providing birds with the required nutrients for maintenance and growth. It is well-known that various feed ingredients have anti-nutritional factors that would either negatively affect digestion, or trap nutrients within their cell wall structure, making them unavailable for the bird to digest and utilize (2, 3) simply because the bird lacks the endogenous enzymes needed to hydrolyze these structures (4, 5). In cereal grains, such as wheat and barley, and in legumes, such as soybeans, the presence of non-starch polysaccharides (NSP) and lignin fiber reduces the extent of digestion and absorption of nutrients by broilers due to increased digesta viscosity (6), as well as encapsulating nutrients within the cell wall structure. Furthermore, birds, especially at a young age, lack the enzymes required to hydrolyze these fibers; arabinoxylan, β-glucan, cellulose, and the non-carbohydrate component lignin are the predominant polymers in cereals. Several commercially available exogenous enzymes have been proven to increase the digestibility of poorly digested cereals to a much greater extent than well-digested cereals (7–10); however, to achieve the maximum improvement in NSP hydrolysis, multiple enzymes may be needed depending on the ingredients used in a diet to hydrolyze the various fibers resulting in an improved energy recovery. Another approach is to feasibly maximize hydrolysis through targeting the most abundant fraction of the NSP in a specific diet by selecting the most effective enzyme for that fiber fraction. Arabinoxylans are the predominant NSP in corn compromising ~5% of the DM (11, 12) and ~50% of the total carbohydrate fraction (13). This fraction can be hydrolyzed by a glycoside hydrolase (GH); the forms most commonly found commercially belong either to the GH10 or GH11 families (14). The GH11 xylanases usually have a larger active site, and they are more substrate specific (14), and degradation produces short-chained oligosaccharide subunits that act as prebiotics for the beneficial bacteria in the gut (15). It has been shown that adding xylanase to poultry diets improves nutrient utilization by increasing its digestibility as well as alleviating the negative effect of many anti-nutritional compounds that the animal cannot digest (16, 17). Furthermore, xylanase has been shown to have a protective effect on the intestinal mucosa barrier to alleviate the negative effect of *Clostridium perfringens* infection in 21 d old broilers (18). However, few studies have focused on the effect of GH11 xylanases on performance and gut health of broilers.

Therefore, the objective of this study was to evaluate the efficacy of different inclusion rates of high efficiency GH11 endo-*1,4*-β-*xylanase*, on live performance, gut lesions, and *Clostridium perfringens* excretion of broiler chickens fed standard corn-soy diets reared under typical broiler production conditions.

## Methods and Materials

Practices conducted during this trial were in compliance with the Guide for the Care and Use of Agricultural Animals in Agricultural Research and Teaching (19).

### Experimental Design

A total of 2,080 Ross 708 mixed sex 1-day-old broilers were obtained from a commercial hatchery and placed in floor pens to evaluate the effect of different xylanase inclusion rates on live performance, severity of gut lesions, and *Clostridium perfringens* excretion of broiler chickens fed standard corn-soy diets. Birds were reared under typical broiler production conditions without any imposed microbial challenge except for what was naturally occurring in the used litter. The experiment was conducted in a completely randomized design and consisted of 5 experimental treatments with 8 replicate floor pens per treatment, each containing 52 chicks and were reared to day 42 of age.

Chicks were reared on used litter topped with fresh pinewood shavings in floor pens (3.05 × 1.52 m) with a minimum of 0.08 m^2^ per bird, provided age-appropriate environmental conditions, and given *ad libitum* access to feed and water. The lighting program included continuous light for the first week (>3 foot candle), then dimmed to 1 foot candle for the remainder of the trial.

### Experimental Diets

Diets were formulated to either meet or exceed the NRC (20) and Ross Broiler Management Handbook (21) requirements for broilers. Three phases were provided: starter (0–21 d), grower (22–35 d), and finisher (36–42 d). The 5 dietary treatments consisted of (1) Positive Control (PC), a standard diet formulated at normal dietary energy level; (2) Negative Control (NC), a standard diet formulated at 130 kcal/kg lower than PC; (3) NC with xylanase at 10 XU/g of feed (NC + 10); (4) NC with xylanase at 12.5 XU/g of feed (NC + 12.5); and (5) NC with xylanase at 15 XU/g of feed (NC + 15). Energy reduction in NC was achieved by adding soy hulls. The xylanase is a GH11 endo-β-1,4-xylanase; a glycosidase that hydrolyzes 1 → 4-β-D-xylosidic linkages in xylan, it is produced by fermentation of a *Komagataella phaffii* microorganism expressing a gene coding for the xylanase enzyme. One unit of endo-1,4- β-Xylanase activity (XU) is defined as the amount of enzyme needed for the release of one nanomole of reduced sugars (xylose equivalents) per second from 0.5% xylan at 50°C in 50 mM trisodium citrate buffer pH 6.0. All diets were corn-soybean meal-based (Table 1). Inclusion levels were consistent at all phases of diet to achieve the activity unit of xylanase in each treatment.

**Table 1:** Composition and nutrient content of experimental diets.

**Ingredient (%)**	**Starter (1–21 d)**		**Grower (22–35 d)**		**Finisher (36–42 d)**	
	**Standard**	**Reduced energy**	**Standard**	**Reduced energy**	**Standard**	**Reduced energy**
Corn	60.75	56.46	67.34	63.08	71.55	67.73
Soybean meal 48%	28.11	27.77	23.30	22.93	14.89	20.90
Poultry meal	5.00	5.00	5.00	5.00	8.24	5.00
Poultry fat	0.05	0.05	0.05	0.05	0.05	0.05
DL-Methionine	0.17	0.18	0.13	0.14	0.02	0.06
Salt	0.48	0.48	0.43	0.43	0.37	0.38
L-Lysine HCl	0.14	0.16	0.15	0.17	0.18	0.08
Limestone	1.5	1.4	1.3	1.2	1.1	1.2
Dicalcium phosphate	2.0	2.0	1.7	1.7	1.6	1.6
Vitamin and mineral premix^1^	0.5	0.5	0.5	0.5	0.5	0.5
Soybean hulls	1.3	6.0	0.1	4.8	1.5	2.5
Calculated nutrients (%)						
Crude protein	22	22	20	20	19	19
Crude fat	1.89	1.84	2.03	1.98	2.49	2.06
Crude fiber	3.02	4.58	2.56	4.12	2.89	3.33
Ash	6.35	6.40	6.64	5.68	5.40	5.42
Calcium	1.05	1.05	0.9	0.9	0.85	0.85
Available phosphate	0.512	0.512	0.45	0.45	0.42	0.42
Sodium	0.22	0.22	0.2	0.2	0.18	0.18
Dig Threonine	0.87	0.88	0.78	0.79	0.68	0.69
Dig Lysine	1.28	1.28	1.15	1.15	1.02	1.02
Dig Methionine + cysteine	0.947	0.947	0.851	0.851	0.755	0.755
Metabolizable energy (kcal/kg)	2,998	2,868	3,100	2,970	3,199	3,069
Analyzed nutrients (%)						
Crude protein	21.25	21.34	19.39	19.47	18.23	18.50
Crude fat	1.44	1.39	1.87	1.84	1.81	2.12
Crude fiber	2.69	4.59	2.38	4.93	4.17	3.53
Ash	7.84	8.81	7.46	7.55	8.77	7.31

1 *Vitamin and trace mineral premix supplied the following per kg of diet: 5,512 IU vitamin A, 1,852 IU vitamin D3, 11 IU vitamin E, 0.06 mg vitamin B12, 0.23 mg biotin, 1.87 mg menadione (K3), 0.44 mg thiamine, 3.75 mg riboflavin, 5.95 mg d-pantothenic acid, 1.32 mg vitamin B6, 34.17 mg niacin and 0.22 mg folic acid, for mineral supplied the following per kg of diet: Manganese: 120 mg, Zinc: 120 mg, Iron: 80 mg, Copper: 10 mg, Iodine, 2.5 mg, Cobalt, 1 mg*.

### Data Collection

#### Live Performance

Live performance measurements were taken at placement and at 21, 35, and 42 days of age. Mortality was recorded daily. Body weight (BW), body weight gain (BWG), feed consumption (FC), feed conversion ratio (FCR) adjusted for mortality, BW coefficient of variation (CV; flock uniformity), and percent mortality were determined.

#### Apparent Metabolizable Energy

At both 19 and 40 d of age, digestibility of apparent metabolizable energy was estimated in four randomly selected birds per pen (two males and two females). Birds were moved to raised-wire cages, and both feed consumption and feces were collected for 3 days. Feces were pooled, processed, and analyzed for dry matter, gross energy, and nitrogen. Feed was also analyzed for dry matter, gross energy, and nitrogen. The following calculations were used to determine apparent metabolizable energy (AME) and nitrogen corrected (AMEn):

$$\begin{array}{l} \text{AMEn}=[\left(\text{FC}\times \text{GEfeed}\right)-\left(\text{DMfecal}\times \text{GEfecal}\right)\\ \;-\left(\text{NR}\times \;8.73\right)]/\text{FC} \end{array}$$

Where FC = feed consumed; GEfeed = gross energy of feed; DMfecal = fecal dry matter; GEfecal = gross energy of feces; NR = nitrogen retention, where NR = (FC × feed nitrogen) − (DMfecal × fecal nitrogen).

#### Intestinal Lesion Score

At 21 and 42 d of age, four birds total (two from each sex) per pen were randomly selected and tested for intestinal lesions in the small and large intestines as an indicator of necrotic enteritis that could have naturally occurred without any challenge. Lesions were scored based on the presence and/or severity of any intestinal lesions using modified method of Dahiya et al. (22). Lesions were scored using a scale from 0 (no lesions found) to 4 (diffused necrosis typical of field cases).

#### Clostridia Perfringens Excretion

Litter samples were analyzed for *Clostridium perfringens* (*C. perfringens*) as an indicator of environmental pathogen load and excretion of *C. perfringens*. Cell forming units of *C. perfringens* (CFUs) per gram of litter was determined prior to placement, at 21 and at 42 days of age following procedure described in FDA BAM, Ch 16 in quadruplicates of 25 grams of litter after homogenizing in 225 ml of peptone diluent (0.1% peptone) and diluting 10-fold, then plating on Tryptose-sulfite-cycloserine agar (TSA) plates. Plates were incubated under anaerobic conditions at ~35°C for ~24 h. Colonies were counted using dilution plates with ~20–200 CFUs. Data was expressed as log_10_ per gram.

#### Statistical Methods

Data were analyzed with one-way ANOVA in a completely randomized design (CRD) with five dietary treatments and eight replicate pens per dietary treatments. The general linear model of Statistical Analysis System (SAS Institute, Cary NC 2017) was employed. Means were separated by LSMEANS. Superscripts were determined based on PDIFF values. Live performance data were analyzed using pen as the experimental unit, while individual birds were considered the experimental units for microbial load and lesion scores with 32 birds per dietary treatment. Means were considered significantly different at *P* ≤ 0.05. The Shapiro-Wilk test was used to calculate the normality of mortality and uniformity data, while the Kruskal-Wallis test was used for analysis of lesion scores.

## Results and Discussion

### Live Performance

Live performance results are shown in Table 2 (FC, BWG, FCR, mortality, and CV of BW). There was no significant difference in FC among treatments; however, BWG increased with the increase in the xylanase inclusion rate from 1 to 42 d. Compared with the NC, the increases in BWG for the xylanase doses of 10, 12.5, and 15 XU/g feed were 34, 71, and 98 g, respectively. The improvement in BWG corresponded to improvement in FCR of 2, 4, and 5 points with adding xylanase to NC at 10, 12.5, and 15 XU/g feed, respectively, compared with NC. The same improvements in broilers at 18 days were reported when different doses of xylanase (0, 1,875, 3,750, and 5,625 XU/kg xylanase) linearly increased BWG and improved FCR (23). Similarly, Olukosi et al. (24) demonstrated that graded levels of xylanase improved broiler growth performance. Saleh et al. (25) reported that feeding low energy diet (90 kcal/kg) reduced BWG, while adding xylanase increased BWG and improved FCR. Similar results were also reported by Nusairat and Wang (17) in broilers. Furthermore, Ravn et al. (26) showed that the addition of a xylanase and arabinofuranosidase combination improved duodenum villi length; an indicator of enhanced growth performance of the broilers. The coefficient of variation (CV) calculation of individual BW data was analyzed at d 0, 21, 35, and 42 of age as an indicator of flock uniformity. All CVs for BW were <10% at 35 and 42 d of age indicating that birds' growth was uniform. Mortality was not affected by treatments. Overall mortality in the entire trial was <3%. As the xylanase activity increased, performance was improved. Digesta viscosity was not measured in this trial; however, this improvement could probably be due to the action of xylanase on degrading the non-starch polysaccharides (NSPs), thus reducing digesta viscosity as well as minimizing the caging effect NSPs have on other nutrients blocking endogenous enzymes from working on them (6, 27).

**Table 2:** Least-squares means for feed consumption (FC), body weight (BW), body weight gain (BWG), feed conversion ratio (FCR), mortality, and BW coefficient of variation for broilers raised to 42 d.

	**Treatments^1^**					**SEM^2^**	***P*-value**
**Age period, d**	**PC**	**NC**	**NC + 10**	**NC + 12.5**	**NC + 15**		
FC, g/bird							
1–21	1,042	1,040	1,044	1,044	1,042	4	0.99
22–35	2,368	2,374	2,364	2,367	2,372	10	0.99
36–42	1,329	1,341	1,342	1,336	1,339	14	0.99
1–42	4,739	4,754	4,750	4,746	4,753	14	0.98
BWG, g/bird							
1–21	806^a^	777^b^	786^a^^b^	798^a^^b^	804^a^	3	0.01
22–35	1,326^a^	1,280^b^	1,291^a^^b^	1,313^a^^b^	1,323^a^^b^	5	0.015
36–42	697	673	687	690	702	6	0.57
1–42	2,829^a^	2,730^c^	2,764^b^^c^	2,801^a^^b^	2,828^a^	8	0.0001
FCR, g:g							
1–21	1.291^a^	1.336^c^	1.322^b^^c^	1.304^a^^b^	1.293^a^	0.004	0.0001
22–35	1.795	1.855	1.836	1.811	1.802	0.008	0.066
36–42	1.751	1.810	1.791	1.780	1.755	0.019	0.84
1–42	1.677^a^	1.736^c^	1.716^b^^c^	1.692^a^^b^	1.682^a^^b^	0.005	0.0001
Mortality, %							
1–21	1.4	1.7	1.7	1.4	1.4	0.3	0.99
22–35	0.1	0.7	0.4	0.1	0.1	0.1	0.59
36–42	0.0	0.3	0.0	0.0	0.0	0.1	0.99
1–42	1.6	2.6	2.1	1.6	1.6	0.3	0.93
Flock uniformity, %							
21	13.7	14.3	13.7	13.7	13.9	0.2	0.55
35	8.5	9.1	9.2	8.8	8.5	0.1	0.05
42	9.1	9.3	9.4	9.4	9.1	0.1	0.71

a−c *means in a row within each variable that lack common superscript differ significantly (P ≤ 0.05)*.

1 *PC, positive control; NC, negative control with ME 130 kcal/kg lower than PC; NC + 10, NC with xylanase at 10 XU/g of feed; NC + 12.5, NC with xylanase at 12.5 XU/g of feed; NC + 15, NC with xylanase at 15 XU/g of feed*.

2 *SEM, standard error of mean for n = 8 pens*.

### Apparent Metabolizable Energy Corrected for Nitrogen (AMEn)

Results for AMEn measured at 21 and 42 d of age are presented in Table 3. Reducing 130 kcal/kg of dietary energy in the NC compared to PC significantly reduced AME and AMEn digestibility of NC. At both 21 and 42 d, xylanase supplementation to NC diets improved AME and AMEn. The AMEn at 42 d of age was improved by 41, 79, and 112 kcal/kg when xylanase was added to NC at 10, 12.5, and 15 XU/g, respectively. These results are in agreement with Liu and Kim (23) who reported that supplementation of different xylanase doses increased gross energy digestibility in broilers. Cowieson and Ravindran (28) also demonstrated that adding an enzyme cocktail that included xylanase at 300 U/kg of feed to broiler diets improved AME. By xylanase enzyme action on the NSPs, additional nutrients, mainly energy, become available for the bird to utilize, leading to improved digestibility.

**Table 3:** Apparent metabolizable energy corrected for nitrogen (AMEn) of broilers raised to 42 d.

	**Treatments^1^**					**SEM^2^**	***P*-value**
**Age, d**	**PC**	**NC**	**NC + 10**	**NC + 12.5**	**NC + 15**		
AME (kcal/kg)							
21	3,034^a^	2,889^e^	2,926^d^	2,973^c^	3,008^b^	9	0.0001
42	3,230^a^	3,078^e^	3,122^d^	3,159^c^	3,191^b^	9	0.0001
AMEn (kcal/kg)							
21	3,004^a^	2,857^e^	2,895^d^	2,942^c^	2,977^b^	9	0.0001
42	3,202^a^	3,054^e^	3,095^d^	3,133^c^	3,166^b^	9	0.0001

a−e *means in a row within each variable that lack common superscript differ significantly (P ≤ 0.05)*.

1 *PC, positive control; NC, negative control with ME 130 kcal/kg lower than PC; NC + 10, NC with xylanase at 10 XU/g of feed; NC + 12.5, NC with xylanase at 12.5 XU/g of feed; NC + 15, NC with xylanase at 15 XU/g of feed*.

2 *SEM, standard error of mean for n = 8 pens*.

### Lesion Score

Table 4 shows that lesion scores at 21 d were similar between PC and NC and were significantly higher than NC + 15 treatment. Lesion scores in general were lower at 42 d, indicating less severe lesions. However, the same trend persisted with NC + 15 having a lower lesion score compared with PC and NC, indicating that the severity of intestinal lesions was not affected by the level of dietary energy but rather by the xylanase level of inclusion. Supplementation of xylanase reduced (*P* = 0.0001) 42-day lesion scores by 13, 14.7, and 24% for NC + 10, NC + 12.5, and NC + 15 compared with NC, respectively. These results are in agreement with the findings of Nusairat et al. (29) that xylanase at 15 XU/g of feed reduced lesion score at 21 and 42 days of age. There was no imposed *C. perfringens* challenge implemented in this trial; however, birds were raised on used litter, which could potentially contain Clostridia and other pathogens. Xylanase helped in ameliorating the negative effect of naturally occurring Necrotic Enteritis probably by mainly reducing the amount of nutrients available to pathogens in the hindgut and environment as well as providing prebiotics of the short units of xylooligosaccharides (XOS) required to support the growth of beneficial bacteria, which ultimately aid in creating a healthy microbiota in the gut.

**Table 4:** Least-squares means for intestinal lesion scores of broilers raised to 42 d.

	**Treatment^1^**					**SEM^2^**	***P*-value**
**Age, d**	**PC**	**NC**	**NC + 10**	**NC + 12.5**	**NC + 15**		
Lesion Score							
21	1.28^b^	1.22^b^	1.13^a^^b^	1.09^a^^b^	0.75^a^	0.05	0.019
42	0.98^b^	0.94^b^	0.82^a^^b^	0.80^a^^b^	0.71^a^	0.02	0.004

a, b *means in a row within each variable that lack common superscript differ significantly (P ≤ 0.05)*.

1 *PC, positive control; NC, negative control with ME 130 kcal/kg lower than PC; NC + 10, NC with xylanase at 10 XU/g of feed; NC + 12.5, NC with xylanase at 12.5 XU/g of feed; NC + 15, NC with xylanase at 15 XU/g of feed*.

2 *SEM, standard error of mean for n = 32 birds*.

### *Clostridia perfringens* Excretion Over Time

**Figure 1:**
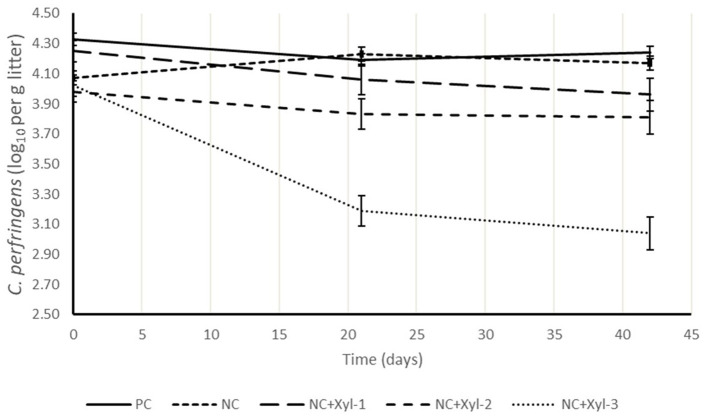
*Clostridium perfringens* in litter samples collected at 0, 21, and 42 d. Experimental diets. ^1^PC, positive control; NC, negative control with ME 130 kcal/kg lower than PC; NC + 10, NC with xylanase at 10 XU/g of feed; NC + 12.5, NC with xylanase at 12.5 XU/g of feed; NC + 15, NC with xylanase at 15 XU/g of feed (0 d: *P* > 0.05; 21 d: *P* ≤ 0.05; 42 d: *P* ≤ 0.05).

The abundance of *C. perfringens* present in litter is shown in Figure 1 and expressed as log_10_(*x*) per gram of litter. Litter samples from each pen were enumerated for its *C. perfringens* counts prior to placement, and at 21 and 42 d. Counts remained relatively constant for PC and NC throughout the trial; however, adding xylanase to NC reduced (*P* < 0.05) *C. perfringens* counts by ~28% compared with control diets. This could be caused by a reduction of nutrients in the hind gut that could be utilized as source of food for pathogen such as *C. perfringens* leading to subsequently improving the intestinal integrity and reducing the lesion score and thus reduce the load in the litter at 21 and 42 days of age as xylanase dose increased.

From these findings it can be concluded that xylanase can improve the nutritional value of corn-SBM based diets, and improve performance, intestinal gut health, and reduce pathogen load in the litter. The concentration of substrate and different feed ingredients in the diet may aid in determining the proper xylanase dose to use. In this study, a supplement of 15 XU/g provided the greatest improvement of body weight gain and lesion scores numerically but was statistically comparable to a dose of 12.5 XU/g. However, energy digestibility improved as the xylanase dose increased. Close attention should be paid to using the right dose of xylanase to optimize economic outcomes, availability of ingredients, and their prices.

## Data Availability Statement

The raw data supporting the conclusions of this article will be made available by the authors, without undue reservation.

## Ethics Statement

The animal study was reviewed and approved by Institutional Animal Care and Use Committee.

## Author Contributions

BN conceptualized and secured funding, designed the study and methodology, organized conducting the trial and collecting data, and performed the data analysis and summary. The manuscript was written by BN and reviewed by both BN and J-JW. All authors have read and agreed to the published version of the manuscript.

## Conflict of Interest

J-JW was employed by the company BioResource International, Inc. The remaining author declares that the research was conducted in the absence of any commercial or financial relationships that could be construed as a potential conflict of interest.
